# *Yersinia pestis* Antigen F1 but Not LcrV Induced Humoral and Cellular Immune Responses in Humans Immunized with Live Plague Vaccine—Comparison of Immunoinformatic and Immunological Approaches

**DOI:** 10.3390/vaccines8040698

**Published:** 2020-11-19

**Authors:** Valentina A. Feodorova, Anna M. Lyapina, Maria A. Khizhnyakova, Sergey S. Zaitsev, Yury V. Saltykov, Vladimir L. Motin

**Affiliations:** 1 Laboratory for Molecular Biology and NanoBiotechnology, Federal Research Center for Virology and Microbiology, Branch in Saratov, 410028 Saratov, Russia; lyapina_anna@inbox.ru (A.M.L.); khizhnyakova_mariya@mail.ru (M.A.K.); zaytsev-sergey@inbox.ru (S.S.Z.); saltykov3443@mail.ru (Y.V.S.); 2 Department of Pathology, Department of Microbiology and Immunology, University of Texas Medical Branch, Galveston, TX 77555, USA

**Keywords:** *Yersinia pestis*, plague, vaccine, immune response, epitope mapping, peptide, immunoinformatics, correlates of immunity

## Abstract

The recent progress in immunoinformatics provided the basis for an accelerated development of target-specific peptide vaccines as an alternative to the traditional vaccine concept. However, there is still limited information on whether the in silico predicted immunoreactive epitopes correspond to those obtained from the actual experiments. Here, humoral and cellular immune responses to two major *Yersinia pestis* protective antigens, F1 and LcrV, were studied in human donors immunized with the live plague vaccine (LPV) based on the attenuated *Y. pestis* strain EV line NIIEG. The F1 antigen provided modest specific cellular (mixed T helper 1 (Th1)/Th2 type) and humoral immune responses in vaccinees irrespective of the amount of annual vaccinations and duration of the post-vaccination period. The probing of the F1 overlapping peptide library with the F1-positive sera revealed the presence of seven linear B cell epitopes, which were all also predicted by in silico assay. The immunoinformatics study evaluated their antigenicity, toxicity, and allergenic properties. The epitope TSQDGNNH was mostly recognized by the sera from recently vaccinated donors rather than antibodies from those immunized decades ago, suggesting the usefulness of this peptide for differentiation between recent and long-term vaccinations. The in silico analysis predicted nine linear LcrV-specific B-cell epitopes; however, weak antibody and cellular immune responses prevented their experimental evaluation, indicating that LcrV is a poor marker of successful vaccination. No specific Th17 immune response to either F1 or LcrV was detected, and there were no detectable serum levels of F1-specific immunoglobulin A (IgA) in vaccinees. Overall, the general approach validated in the LPV model could be valuable for the rational design of vaccines against other neglected and novel emerging infections with high pandemic potency.

## 1. Introduction

To prevent the global spread of emerging infection diseases (IDs), effective and innovative vaccine strategies are needed, particularly for the eradication of especially dangerous, neglected, and novel IDs. In fact, during the last two centuries, active vaccination resulted in a significant reduction in morbidity and mortality caused by a number of IDs. Currently, vaccination saves millions of lives annually worldwide [1]. Progress in vaccine research has provided a basis for the implementation of different classes of vaccines based on live-attenuated or killed pathogens, inactivated toxins, vector carriers expressing desired antigens, recombinant and synthetic peptides, adjuvants, glycoconjugates, nucleic acids, and other strategies. [1]. Nevertheless, the inevitable and unpredictable emergence or re-emergence of IDs, especially IDs with high epidemic and pandemic potency, present a variety of global challenges for human and animal health. Therefore, innovative technologies are needed to shorten the period of vaccine development in order to accelerate and significantly improve vaccine design, especially when a novel ID suddenly emerges. In this respect, the progress in molecular biology in combination with immunoinformatic tools is the driving force in the vaccine design process toward the development of next-generation effective and safe vaccines with improved immunogenicity. Computational vaccinology provides precise and fast epitope mapping of target antigens to identify immunogenic and nonimmunogenic, as well as immunoreactive and nonimmunoreactive, protein component(s), resulting in the selection of peptides consisting of a few amino acids [2]. In fact, this approach can accurately identify a limited number of candidate peptides with predicted marked antigenic and nonallergenic activity among individual immunoreactive epitopes. Overall, computational vaccinology can reduce or significantly minimize the adverse effects of vaccination and provide a wide availability of epitope-based vaccines, which present a rational alternative to the conventional vaccine design concepts [3,4,5]. Nevertheless, the computational identification of specific individual immunoreactive immunodominant epitopes on the target protein still requires confirmation of their efficacy in vivo. In this regard, a comparison of in silico predicted immunoreactive epitopes with those mapped after actual human vaccination can be useful in selection of the best peptide candidate to enter the efficacy studies. One such possibility arises from the use of a vaccine with a long history of application and documented immunity in vaccinees. The study of the immune response in the cohort of vaccinated donors compared with the analysis generated by the immunoinformatics tools in parallel could clarify the epitope specificity of the immune response to the target immunodominant proteins.

From our perspective, the live whole-cell (LWC) plague vaccine (LPV) can serve as a valuable model for the selection of vaccine candidate peptides on the basis of both direct human vaccination studies and immunoinformatics. Vaccination with the *Yersinia pestis* strain EV line NIIEG, the derivative of the French EV strain, leads to the development of self-limited infection that mimics the initial stage of the plague disease and provides natural presentation of the major antigens expressed in vivo [6]. Since the early 1930s, the LPV was successfully used for immunization of both humans and animals with more than 10 million people vaccinated in the Former Soviet Union (FSU) alone without vaccine-related death or dramatic post-vaccinated adverse effects [6]. Inoculation of LPV elicited a high level of protection against pneumonic and bubonic plague although this vaccine is now licensed for human use only in Russia and some FSU countries [6].

Two key antigens of interest, i.e., the capsular antigen F1 and the virulence antigen LcrV, are the major components of practically all plague vaccines under development [7]. Both antigens, in different vaccine formulations, induced a high level of protection in small animal models accompanied by specific antibody immunoglobulin G (IgG) responses determined by ELISA. Many such formulations contained different fusion peptides between F1 and LcrV or their parts with other proteins, indicating the possibility of a further reduction in size of these protective antigens [8,9,10,11,12,13,14,15,16].

Recently, a combined high-throughput computational and experimental effort toward the identification of cluster of differential 8 (CD8) T-cell epitopes for *Y. pestis* has been reported [17,18,19,20]. The main goal of our study was to investigate the immune response to F1 and LcrV in humans immunized with LPV followed by B-cell epitope mapping using a library of synthetic peptides and immunoinformatic tools for in silico prediction of the individual linear and conformational epitopes for these proteins.

## 2. Materials and Methods

### 2.1. Ethics Statement

The study was approved by the Institutional Review Board of the State Educational Institution of Higher Professional Education, Saratov Medical State University (IRB#00005197) and followed the rules stated in the Declaration of Helsinki. Each human volunteer provided written informed consent for blood donation and subsequent use of the samples for research goals, as well as publication of the relevant case details.

### 2.2. Study Subjects

The study cohort (group A-Total) consisted of 34 healthy donors aged from 26 to 72 years of both genders who received different numbers of annual immunizations (1–51 injections) with the live plague vaccine *Y. pestis* line EV NIIEG (LPV). The control group (group B) included healthy 17 individuals who had no history of contact with either *Y. pestis* microbe or its antigens. The group A-Total of immunized donors was also divided into subgroups of recently vaccinated (A-RV, less than 1 year post vaccination, *n* = 14) and early vaccinated (A-EV, more than 1 year post vaccination, *n* = 20) donors. The vaccination was performed by experienced medical workers to plague researchers via intradermal immunization (scarification) according to the standard procedure for vaccination with LPV in Russia [6].

### 2.3. Y. pestis Recombinant Antigens

*Y. pestis* recombinant proteins LcrV and F1 were cloned and expressed in *Escherichia coli* as a C-terminal fusion with a polyhistidine tag, followed by affinity purification using Ni^2+^- chromatography as previously described for the panel of five antigens [21]. The quality of purification was evaluated with the silver-stained PAGE technique. Both antigens were essentially lipopolysaccharide (LPS)-free, as assessed with the QCL-1000 Chromogenic LAL Assay kit (Fisher Scientific, Waltham, MA, USA).

### 2.4. Isolation of PBMCs, Proliferation Assay, and Cytokine Profiling

The procedure for PBMC isolation, proliferation assay, and cytokine quantification was previously described [22]. Briefly, PBMCs were isolated from heparinized blood by density gradient centrifugation according to a standard protocol and seeded immediately at a concentration of 10^5^ cells per well. Cells were cultured in Dulbecco’s modified Eagle medium (DMEM)/F12 medium supplemented with 10% fetal bovine serum (FBS) and antibiotic–antimycotic solution in 96-well plates for 6 days with or without recombinant F1 or LcrV (both at 5 μg/mL) or concanavalin A (Sigma, North Liberty, IA, USA) at 1 μg/mL, used as a positive stimulation control. The proliferative response was measured using a Cell Proliferation ELISA, BrdU chemiluminescent kit (Roche Diagnostics, Mannheim, Germany) according to the manufacturer’s protocol. Stimulation indices (SIs) were calculated by dividing the mean relative light units per second (rlu/s) obtained for the cultured cells with a stimulant by the rlu/s of nonstimulated wells.

On day 5, supernatants of cell cultures were collected and preserved at −80 °C for further cytokine detection. Commercial ELISA kits were used for the measurement of interferon (IFN)-γ, tumor necrosis factor (TNF)-α, interleukin (IL)-4, IL-10, and IL-17A levels (Vector-Best, Koltsovo, Russia; Cytokine, St. Petersburg, Russia).

### 2.5. Serum Antibody Detection

The basic protocols utilized for detection and quantitative antibody analyses were previously described [22]. For quantification of specific antibody titers, ELISA was used. Briefly, Immulon 2 HB plates (Thermo Scientific, Waltham, MA, USA) were coated with the recombinant F1 or LcrV antigen at a concentration 2 μg/mL dissolved in 0.1 M carbonate buffer, pH 9.5, and incubated overnight at 4 °C. Newborn calf serum (Sigma, North Liberty, IA, USA) at concentration of 20% in phosphate-buffered saline (PBS) was used for both blocking and dilution of sera samples. Bound antigen-specific antibodies were detected using horseradish peroxidase (HRP)-coupled goat anti-human IgG (Sigma, North Liberty, IA, USA). The reaction was developed with the 3,3’,5,5’-tetramethylbenzidine (TMB) substrate (Sigma, North Liberty, IA, USA) and read by the Synergy HT Microplate Reader (BioTeK, Winooski, VT, USA). The titers were calculated as the last sample dilution giving a signal above the cutoff level that was the twice of mean value of the blank wells (sera without antigen).

Qualitative antibody analysis was performed using the immunoblotting technique. Antigens were transferred to a 0.22 μm pore size nitrocellulose membrane (Bio-Rad, Hercules, CA, USA) after SDS-PAGE in 12.5% (LcrV) or 15% (F1) acrylamide gel and incubated with corresponding human sera at the dilution 1:200 overnight at 4 °C. Peroxidase-conjugated goat anti-human IgG (Sigma, North Liberty, IA, USA) was used as a secondary antibody. The reaction was developed with the TMB substrate for the membranes (Sigma, North Liberty, IA, USA). Blots were analyzed visually. The excess of the color signal of the antigen–antibody binding reaction over the background level was considered as a positive reaction.

### 2.6. Antibody Isotyping

Detection of immunoglobulin isotypes was performed using either ELISA or immunoblotting techniques. All steps prior to the conjugate addition are described in the previous section. Commercial murine monoclonal antibodies directed against human Ig classes (IgM, IgA, IgE), and IgG subclasses (IgG1, IgG2, IgG3, IgG4) (Rosmedbio Ltd., St. Petersburg, Russia) were added after incubation with serially diluted human sera. Goat anti-mouse IgG (Fab-specific)-HRP Ab (Sigma, North Liberty, IA, USA) was used as secondary antibody. The reaction was developed with the TMB substrate for either membrane or ELISA and read with the relevant methods described above.

### 2.7. In Silico Prediction of Linear and Conformational B-Cell Epitopes for F1 and LcrV

Both potential linear and conformational B-cell epitopes for either F1 or LcrV were predicted by ElliPro (http://tools.iedb.org/ellipro/) [23] on the basis of the relevant protein sequences and three-dimensional (3D) models for each antigen. The ElliPro service was also used for the development of ribbon diagram models of the 3D structures of the individual predicted immunoreactive protein linear and conformational epitopes for both antigens.

### 2.8. Allergenicity and Toxicity Prediction

The allergenicity was predicted using AllerTOP v2.0 (http://www.ddg-pharmfac.net/AllerTOP) [24]. The toxicity was evaluated using the ToxinPred (http://crdd.osdd.net/raghava/toxinpred) web server [25]. The antigenicity prediction was uncovered using the VaxiJen v2.0 server (http://www.ddg-pharmfac.net/vaxijen/VaxiJen/VaxiJen.html) with the FASTA formatted amino-acid sequences of entire proteins [26].

### 2.9. Identification of F1 Immunoreactive Linear B-Cell Epitopes Using Epitope Mapping

The protocol for B-cell epitope mapping ELISA was described by us previously [22]. A library of 27 synthetic peptides (Genscript, Piscataway, NJ, USA) covered the full length of the F1 antigen generated from the F1 sequence of *Y. pestis* CO92 (accession no. NP_395430.1) and consisted of 14- to 17-mer peptides with 11-amino-acid overlaps. The library composition can be found in Table S1 (Supplementary Materials). Briefly, Nunc Immobilizer, Amino Modules Plates (Thermo Scientific, Waltham, MA, USA) were coated with 20 μg of individual peptides diluted in 0.1 M carbonate buffer, pH 9.5, and incubated overnight at 4 °C. All subsequent steps of analysis were identical to the protocol described for serum antibody detection with tested sera loaded in dilution of 1:100. Absorbance was measured at 450 nm by BioTek Synergy HT reader(BioTeK, Winooski, VT, USA). A signal was considered positive when it reached the cutoff value of twice the background optical density (OD) which was calculated as the mean of the lowest 50% of all OD values obtained for all peptides and control wells with that particular serum [23]. We used wells without peptides as negative controls and wells containing recombinant F1 as a positive control.

### 2.10. Statistical Analysis

GraphPad Prism 6.01 (GraphPad software, San Diego, CA, USA) was used for data analysis and graphic representation. The results were expressed as min to max for the cytokine and proliferation assays. For antibody assessment, geometric mean titers (GMTs) were calculated. For this purpose, donors with antibody titers below the limit of detection were arbitrarily assigned a value of half the initial dilution. The Wilcoxon’s signed-rank test was used for analysis of differences between groups for paired samples, the Mann–Whitney test was applied for independent samples, and the Fisher’s exact test was used in the case of dichotomous variables. Receiver operating characteristic (ROC) analysis was performed for evaluation of anti-F1 antibody titers as a marker of vaccination with LPV. Associations were assessed using Spearman’s Rank Correlation coefficient. A *p*-value <0.05 was considered significant.

## 3. Results

### 3.1. Proliferative and Cytokine Response to In Vitro Antigen Stimuli

**Figure 1:**
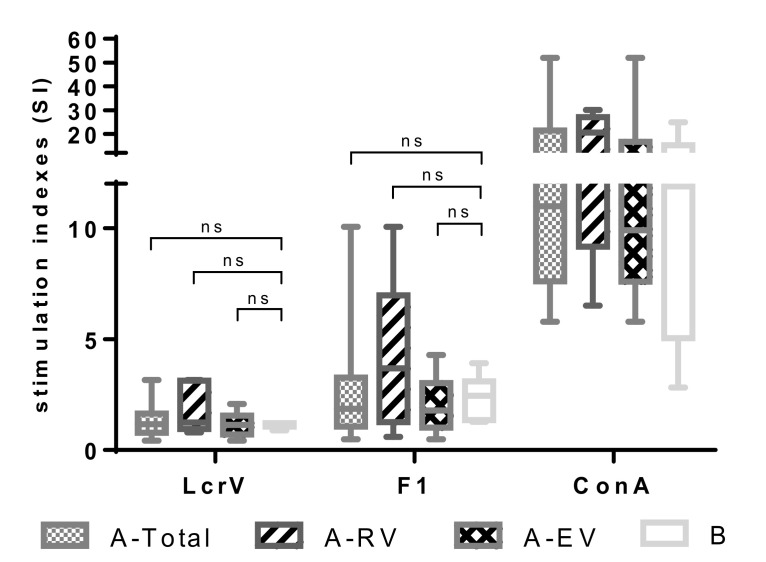
Proliferative response of peripheral blood mononuclear cells (PBMCs) obtained from vaccinated with live plague vaccine (LPV) (A-Total, A-recently vaccinated (RV), A-early vaccinated (EV)) and unvaccinated (group B) donors induced to in vitro F1 and LcrV stimuli. PBMCs were stimulated with either a recombinant F1 or LcrV (both at 5 μg/mL), or concanavalin A (ConA; 1 μg/mL) as a positive control. Stimulation indices were calculated as mean counts of quadruplicates in antigen-stimulated cultures divided by the mean counts of quadruplicates in unstimulated cultures. Results are expressed as min to max (whiskers) with the median designated as a horizontal line, and the box extends from 25th to 75th percentiles. The Mann–Whitney test was performed for statistical analysis; ns: not significant.

The cellular immune response to F1 and LcrV elicited by the LPV was first assessed by studying the proliferative responses of peripheral blood mononuclear cells (PBMCs) from vaccinated versus naïve donors. PBMCs were stimulated with 5 μg/mL of recombinant F1 or LcrV. Concanavalin A (Con A, 1 µg/mL) served as a positive control stimulus. The number of samples used was *n* = 18 for A-Total, *n* = 5 for A-RV, *n* = 13 for A-EV, and *n* = 6 for group B. To our surprise, in vitro stimulation with either LcrV or F1 did not induce marked proliferation of lymphocytes from all the groups of LPV-vaccinated donors. No significant difference was observed in response to both antigen stimuli compared to the unvaccinated control group (Figure 1). Indeed, the median SI values obtained after stimulation with LcrV were extremely low and did not exceed 1.25 (A-RV). Although a moderate trend of higher SI to F1 stimulus, particularly in recently vaccinated donors (A-RV, median SI value, 3.68), was observed, this difference was also not significant (*p* > 0.05).

**Figure 2:**
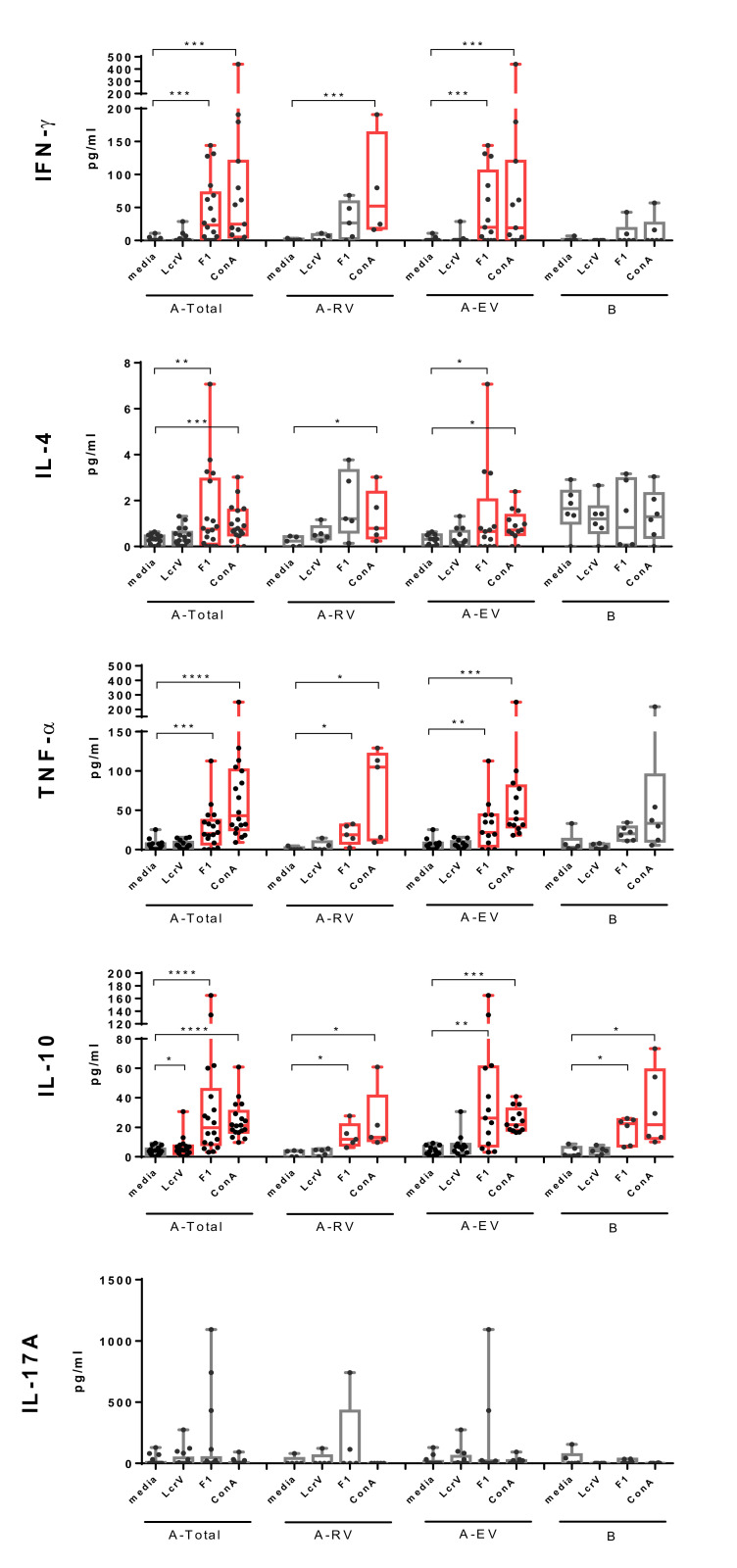
Levels of interferon (IFN)-γ, interleukin (IL)-4, tumor necrosis factor (TNF)-α, IL-10, and IL-17A detected in supernatants of PBMCs from vaccinees vaccinated with LPV (A-Total, A-RV, A-EV) and unvaccinated naïve donors (group B) stimulated with recombinant F1 or LcrV, or ConA as a positive control. Results are expressed as min to max (whiskers) with the median designated as a horizontal line, and the box extends from the 25th to 75th percentiles. The Wilcoxon’s signed-rank test was used for statistical analysis of difference between spontaneous (media) and stimulated secretion of cytokines (significant differences are designated in red color), while the Mann–Whitney test was performed for comparison of unpaired data; * *p* < 0.05, ** *p* < 0.01, *** *p* < 0.001, **** *p* < 0.0001. Significantly increased cytokine levels are colored in red.

Cellular immune responses against F1 and LcrV were further tested by monitoring cytokine production in supernatants of stimulated PBMCs (Figure 2). Re-stimulation with F1 induced marked secretion of a panel of pro- (IFN-γ, TNF-α) and anti-inflammatory (IL-4) or regulatory (IL-10) cytokines. Importantly, F1-induced production of IFN-γ, IL-4, and TNF-α was specific for LPV immunization, since it was detected in PBMCs obtained from LPV-vaccinated donors only. The increased levels of IFN-γ and IL-4 were predominantly registered in early vaccinated group (*p* < 0.05). Although the same trend was observed in recently vaccinated donors, it did not reach statistical significance (*p* > 0.05), probably due to the small number of vaccinees in the A-RV group. Overall, the increase in secretion of IFN-γ and IL-4 in response to F1 stimulus in PBMCs from vaccinated donors (A-Total) was comparable to that induced by the positive control Con A, while TNF-α levels were lower (*p* < 0.05). F1-induced production of IL-10 was also marked, but was detected in both vaccinated and unvaccinated individuals, likely indicating that there was no connection between memory recall IL-10 response and the LPV immunization.

In contrast, stimulation with LcrV induced increased levels of IL-10 in PBMCs obtained from vaccinees only. A significant difference between unstimulated and stimulated cytokine production was observed in the A-Total group (*p* < 0.05), while there was no statistically significance difference for groups A-RV and A-EV in this category (*p* > 0.05).

Interestingly, neither F1 nor LcrV stimuli affected IL-17A production in either the vaccinated or the unvaccinated group of donors, probably due to the low frequency of IL-17A-producing cells in peripheral blood samples.

Associations among proliferative response, cytokines levels, and number of received vaccinations with the LPV or duration of post vaccination period were also evaluated. No positive correlation was observed for all tested cytokines, as well as for antigen-stimulated proliferation (SI) (*p* > 0.05) (Figures S1 and S2, Supplementary Materials).

### 3.2. Humoral Immune Response to F1 and LcrV in Vaccinees

**Figure 3:**
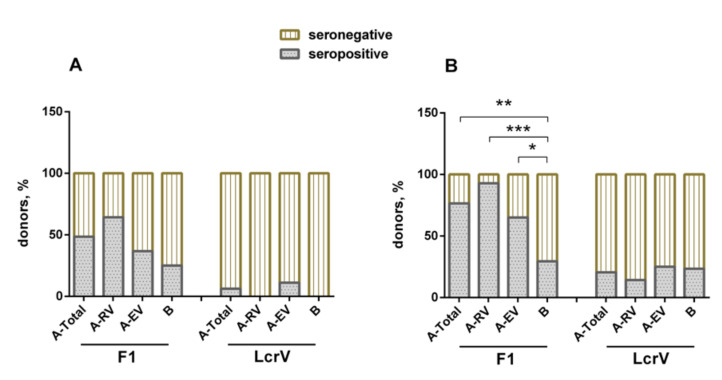
Detection of antigen-specific antibodies to F1 and LcrV in sera obtained from vaccinees (groups A-Total, A-RV, A-EV) and naïve donors (group B) using (**A**) ELISA and (**B**) the immunoblot technique. Fisher’s exact test was used for analysis of difference between groups; * *p* < 0.05, ** *p* < 0.01, *** *p* < 0.001.

The individual humoral immune response to either F1 or LcrV induced by LPV was assessed in vaccinees and naïve volunteers as described by us previously [22]. The ELISA results revealed that the percentage of formally seropositive donors to F1 varied insignificantly among the groups, such as 25% in the unvaccinated control group B, and 48.5%, 36.8%, and 64.4% in vaccinees for the groups A-Total, A-EV, and A-RV, respectively (Figure 3A). Interestingly, only two vaccinated individuals from the A-EV group revealed the presence of specific antibodies to LcrV, while the A-RV and B groups did not have positive reactions. The antibody titers to F1 and LcrV detected in the sera of both vaccinated and unvaccinated donors are shown in Figure S3 and summarized in Table S2 (Supplementary Materials). Only group A-RV demonstrated statistically significant geometric mean antibody titers to F1 with a large variation in the titer’s values (100.0, 95% confidence interval (CI), 45.6–219.2). The titers to F1 in the sera of unvaccinated volunteers were relatively low (33.9, 95% CI, 25.1–45.7). No statistically significant differences in GMT to LcrV in sera of any groups were found.

In general, the immunoblot technique showed results that were very similar to those obtained with ELISA, but with an increased number of positive donors in all groups. Importantly, the difference between vaccinated and naïve donors became statistically significant for the F1 antigen response. The majority of vaccinees (26/34, 76.5%) revealed a specific antibody response to F1 (*p* < 0.01), whereas only a few of them (7/34, 20.6%) demonstrated the presence of antibody to LcrV, with no significant difference in anti-LcrV positive responses between vaccinated and unvaccinated donors (*p* > 0.05) (Figure 3B).

Vaccinees in both groups, A-EV (*p* < 0.05) and A-RV (*p* < 0.001), showed specific positive reactions to F1, and there were more (*p* > 0.05) positive donors among the recently vaccinated donors (13/14, 92.9%) in comparison with those who received their immunizations early (13/20, 65.0%). As seen with the ELISA results, the vaccinees developed a poor response to LcrV in both A-EV (5/20, 25%) and A-EV (2/14, 14.3%). In both cases, there was no significant difference compared to group B unvaccinated donors (Figure 3B). Nevertheless, there were four donors in the A-EV and two donors in A-RV groups who simultaneously had antibodies to both F1 and LcrV (data not shown).

There was a slight negative correlation between the number of immunizations with LPV and the serological response to either F1 or LcrV, although no statistical significance was reached for both antigens (Figures S4a and S5a, Supplementary Materials). Furthermore, there was a slight negative correlation between the number of years post vaccination and antibody response to LcrV. On the contrary, F1 demonstrated a minor positive correlation in this category, albeit not statistically significant (Figures S4b and S5b, Supplementary Materials).

The presence of specific antibodies to F1 of the IgM, IgG, IgA, and IgE classes was observed in both vaccinated and unvaccinated donors with exception to IgE in group B. No significant difference between the groups was detected (*p* > 0.05). Importantly, all vaccinees (group A) possessed anti-F1 antibodies for IgG1–IgG4 subclasses, while naïve donors of the group B possessed F1-specific antibodies of only two IgG subclasses, namely, IgG1 and IgG3 (Figures S6 and S7, Supplementary Materials). Nevertheless, the ELISA antibody titers to F1 in vaccinees were IgG1 > IgG2 (Figure S6. Supplementary Materials). Unfortunately, it was not possible to subtype the antibody to LcrV due to the low titers of the relevant antibody (Figure 3B).

For assessment of a diagnostic value of anti-F1-antibody titers for discrimination between LPV vaccinated and unvaccinated donors, receiver operating characteristic (ROC) analysis was performed. An area under the curve (AUC) = 0.7338 was obtained for ROC analysis of anti-F1 titers between groups A-RV and B (*p* < 0.05) (Figure S8, Supplementary Materials); however, for groups A-Total and B, no statistical significance was found (*p* > 0.05) (Figure S9, Supplementary Materials). Nevertheless, the results indicated that F1-specific antibodies have the potential to serve as an immunological marker of vaccination with LPV at least in donors who received immunization during the 1 year period prior to sera sample collection. The cutoff value for antibody titers detected in ELISA given the highest Youden’s index was 150. Although the specificity of the test calculated in this case was absolute (100%), the sensitivity was quite low (42.86%). This result makes questionable the significance of anti-F1 antibody titers as an overall effective marker of immunization success with LPV in humans.

### 3.3. In Silico Prediction of B-Cell Linear Epitopes of F1 and LcrV

On the basis of the ElliPro analysis, a total of seven sequences were predicted as potential linear B-cell epitopes on F1 and nine sequences on LcrV (Table 1). The epitopes were numbered sequentially through each of the antigens, i.e., from 1–7 for F1 and from 1–9 for LcrV. All seven F1 epitopes were antigenic, nontoxic, and exposed on the protein surface (Figure S10a, Supplementary Materials). However, only three out of seven epitopes (epitopes 2, 3, and 7) were predicted to be nonallergenic, while the remainder (epitopes 1, 4, 5, and 6) were assigned as probable allergens. Thus, epitopes 2, 3, and 7 with a length in the range of 7–16 amino-acid residues could be potential linear B-cell epitopes useful for vaccine design if they appear in the experimental host immune response in vaccinees.

Similarly, no toxic epitopes were found in the LcrV molecule and all of them were located on the surface of the antigen (Figure S10b, Supplementary Materials). In fact, five out of nine epitopes (epitopes 2, 4, 5, 8, and 9) were predicted as possessing antigenic activity. However, only two of them (epitopes 8 and 9) appeared to be nonallergenic, while four out of nine epitopes (epitopes 2, 4, 5, and 6) showed a high probability to induce allergic reactions. Three epitopes, numbers 1, 3, and 7, were determined to be nonantigenic and nonallergenic.

Furthermore, discontinuous or conformational B-cell epitopes were predicted for F1 (three epitopes) and LcrV (five epitopes) using the ElliPro tool of IEDB. The individual residues, residue position, length, and the scores determined in this study are presented in Table 2. According to the data predicted by the ElliPro, the linear epitopes predicted were involved in the formation of the conformational B-cell epitopes. According to the model for 3D structures of the individual predicted immunoreactive protein conformational epitopes for both antigens, all of them were located on the relevant protein surfaces (Figure S10b, Supplementary Materials).

### 3.4. Epitope Mapping of F1 Antigen with the Use of Overlapping Peptide Library

**Figure 4:**
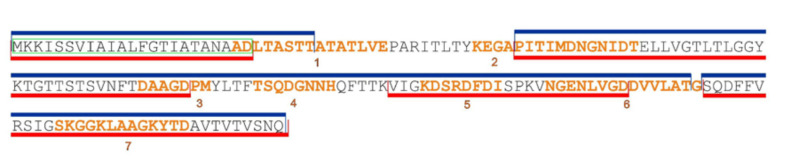
In silico identification of seven linear epitopes predicted by ElliPro (http://tools.iedb.org/ellipro/) [23]. The epitopes are numbered sequentially from 1–7 through the F1 protein and colored in orange. The fragments of F1 positive in ELISA with peptide library tested with sera from the vaccines of the groups A-RV and A-EV are colored in blue and red, respectively. The green box indicates a signal peptide on F1 full-length sequence.

**Figure 5:**
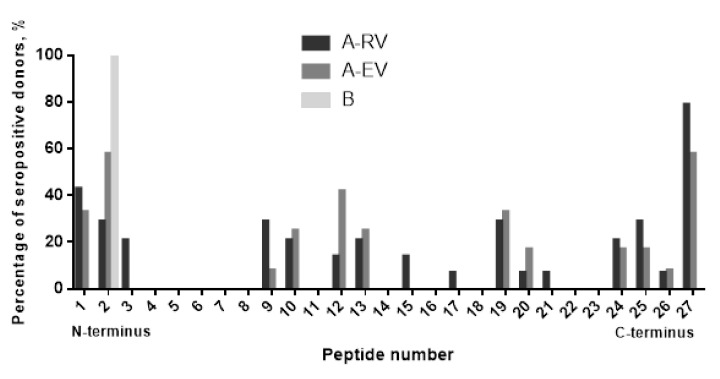
F1 antigen B-cell epitope mapping with sera from LPV-vaccinated donors. A library of 27 peptides designed as 14- and 17-mers with 11 overlaps was used to identify immunoreactive epitopes against sera from donors vaccinated with LPV with different post-immunization periods (A-RV and A-EV groups). The only serum suitable for peptide analysis among the sera with the positive reaction to recombinant F1 in ELISA of the group B (unvaccinated individuals) reacted with peptide 2 corresponding to the part of the leader amino-acid sequence. A signal was considered positive when it reached the cutoff value of twice the background optical density (OD). The background OD was the mean of the lowest 50% of all OD values obtained for all peptides and control wells with that particular serum as described by us previously [22].

To validate the epitopes predicted in silico, the sera of vaccinees with the highest titers to F1 were tested by ELISA with a library of 27 overlapping 14- and 17-mer peptides offset by 11 residues generated from the F1 full sequence. We found that all seven predicted linear epitopes were immunoreactive. However, there was a certain difference in how sera from A-RV and A-EV groups of vaccinated donors reacted with the peptide library. The sera from recently vaccinated donors of A-RV group (*n* = 12 tested) interacted widely with almost all of the F1 molecule, covering partially or the entire (all seven) linear epitopes predicted (Figure 4). In contrast, the sera from the early vaccinated group A-EV (*n* = 14 tested) were more specific to certain regions of F1, resulting in positive reactions with five out of seven epitopes predicted, namely, epitopes 2, 3, 5, 6, and 7. The sera from both A-RV and A-EV groups provided nonspecific positive reactions with the hydrophobic leader sequence peptide of F1, which served as an internal control for the ELISA. Overall, 16 out of 27 peptides in the library were immunoreactive, such as peptides 1–3, 9, 10, 12, 13, 15, 17, 19–21, and 24–27. All of them reacted with the sera from the A-RV group, and, with the exception of peptides 3, 15, and 17, all of them reacted with the sera from A-EV group. Only peptide 2 had a positive reaction with the negative control serum from an unvaccinated donor from group B (Figure 5).

Summarizing the analysis of both in silico epitope prediction and actual response in vaccinees to F1 antigen, we found that three linear epitopes (numbers 2, 3, and 7) predicted to potentially possess both antigenic and nonallergenic activity were confirmed to be involved in the humoral immune response in human subjects. Three epitopes (numbers 4–6) with probable allergenic activity also demonstrated their ability to induce the homologous specific antibody in humans after immunization with LPV. In fact, epitope 4 was reactive with antibodies produced after a single injection with LPV. Epitope 1 provided a cross-reaction with the sera from unvaccinated individuals. Unfortunately, it was not possible to validate the immunoreactive epitopes for LcrV due to the low antibody titers to this antigen in sera of the donors vaccinated with LPV (Figure 3B).

## 4. Discussion

One of the major points of this research was to investigate whether the computational vaccinology approach is actually valuable in predicting the immune potency at a single epitope level for the immunodominant and protective *Y. pestis* antigens F1 and LcrV. If true, data obtained from this study could be crucial for the development of highly effective and safe peptide vaccines capable of inducing long-term and robust immune responses against the plague pathogen for human and veterinary applications. Additionally, the value of each antigen as an immunological marker of response to human vaccination with LPV was studied. Finally, in silico immunoinformatic tools were applied for searching both linear and conformational B-cell epitopes within F1 and LcrV sequences, which are involved in the LPV-mediated immune response in vaccinees.

It is well established that active and passive immunizations with recombinant F1/LcrV- based vaccine candidates provide a high level of protection against both bubonic and pneumonic plague in small animal models while demonstrating variable levels of protective immunity in nonhuman primates [7,28]. Moreover, both F1 and LcrV antigens were identified among a set of the major antigens which were recognized by murine and human convalescent sera during the course of experimental plague infection [29,30,31]. Therefore, both antigens should be actively involved in the immune response induced in humans during vaccination with the attenuated strain of *Y. pestis* EV line NIIEG used in Russia as a live plague vaccine [6].

As expected, the F1 antigen provided modest specific cellular and humoral immune responses in the majority of vaccinees independently from the number of annual vaccinations with the LPV, as well as from the duration of the post-vaccination period. The discrepancy in the overall number of donors seroconverted to F1 determined with ELISA versus immunoblotting (Figure 3A,B) could be attributed to the differences in the methods of detection distinguished in the recognition of conformational and linear epitopes. Furthermore, computational analysis of the F1 amino-acid sequence showed that all epitopes predicted in silico (Table 1 and Table 2) were surface-exposed (Figure S10, Supplementary Materials), i.e., were ready to interact with and prime the immune system of the vaccinees. The actual antigenicity of these F1 epitopes predicted by immunoinformatics was experimentally confirmed with the use of a panel of the F1-positive sera obtained from vaccinees. In fact, all seven epitopes predicted for F1 were immunoreactive and revealed a positive reaction in the immunoassay (ELISA) with the F1 peptide library (Figure 4). Moreover, all seven epitopes identified in this study were recognized by the serum anti-F1 antibodies derived from recently vaccinated donors, while five out of seven epitopes (number 2, 3, 5, 6, and 7) preserved their immunoreactivity for more than 30 years after the last immunization with LPV. In contrast, the epitope TSQDGNNH (number 4) was recognized by the anti-F1 antibodies from the sera of recently vaccinated donors only, suggesting that this peptide can be potentially used for differentiation between recent and long-term vaccinations. Interestingly, the antibody response to F1 was revealed in some naïve unvaccinated donors. This result conforms with the early observations obtained both in animals [32] and humans [28]. However, we observed that at least one peptide across the F1 sequence was reactive with sera obtained from naïve donors (Figure 5). This peptide corresponded to the terminal part of the leader amino-acid sequence, and this finding could explain the positive reactions in F1-ELISA with sera from nonimmune donors. Notably, all epitopes predicted and verified in our study generally corresponded to the F1 immunoreactive epitopes revealed early in animal models, suggesting their universal nature [18,20,33,34,35,36]. Overall, the presence of many highly reactive epitopes within F1 could explain the high antigenic activity of this protein capable of generating the relevant specific B-cell response in vaccinees that exist for up to 50 years (observation period of our study), providing the persistence of anti-F1 antibodies for such a long period of time.

Indeed, the antibody to F1 could be detected in the sera of vaccinees more than 30 years later (Figure S2, Supplementary Materials). This is longer than that measured in patients that recovered from natural plague infection, as these survivors showed the persistence of F1 antibody for more than a decade [30]. However, there was a clear decrease in the number of seropositive responders over time (1.8- and 1.5-fold reductions detected by ELISA and immunoblotting, respectively) following the last injection with LPV. This observation was in correlation with that reported by us earlier [37]. This could be explained by the persistence of anti-F1 antibodies that recognize only a few F1 epitopes (Figure 4). The immunoinformatics approach further identified four out of seven F1 epitopes as having possible allergenic activity (Table 1), which could potentially be involved in the development of post-vaccinal LPV-associated side effects reported earlier [6,7].

In summary, immunoinformatics and computational simulation tools resulted in the identification of three F1 epitopes, namely, KEGAPITIMDNGNIDT, DAAGDPM, and SKGGKLAAGKYTD (numbers 2, 3, and 7), that could serve as potential peptide vaccine candidates. These three epitopes were antigenic, nontoxic, and nonallergenic and, upon LPV vaccination, were able to promptly induce specific antibodies to F1, which then persisted for a long timeframe in vaccinees.

The earlier immunized individuals from the group A-EV demonstrated a F1-specific cellular recall reaction (*p* < 0.05) that was observed in some vaccinees even after more than two decades since the last injection with LPV (Figure 2; Figure S2, Supplementary Materials). This means that, in contrast to convalescents formed after an acute plague in humans and animals, immunization with LPV elicited long-lasting B and T memory cells in vaccinees [29,30,38,39]. Nevertheless, a marked variability in F1-specific immune response in vaccinated individuals raises doubts about a possible leading role of the F1 antigen alone as the main marker of correlates of protective immunity for LPV vaccination, especially for evaluation of the long-term humoral immune response and retrospective analysis of LPV potency. At the same time, ROC analysis provided statistical evidence of a potency of antibody response to F1 alone during an early post-vaccination period (Figure S9, Supplementary Materials).

Interestingly, F1 could induce a mixed T helper 1 (Th1)/Th2 type of immunity in vaccinees, as indicated by the significantly elevated TNF-α, IFN-γ, and IL-4 levels upon stimulation of PBMCs derived from the early vaccinated donors (Figure 2). Nevertheless, a marked prevalence of IgG1 over IgG2 (Figure S6, Supplementary Materials) likely indicates an immune response polarization mainly toward Th2. The distinguished T cell recall in vaccinees may reflect a difference between the mechanisms of immune response to LPV versus convalescents formed after an acute plague who demonstrated no cellular immune response to F1 [30]. Another explanation can be attributed to the variations in experimental procedures for evaluation of the cellular response between the studies. The critical importance of the choice of methods used for the assessment of cell-mediated immunity was recently illustrated in studies of the immune response elicited by Flagellin/F1/V candidate vaccine [40,41].

Importantly, the immunization with LPV did not elicit a detectable cellular response and provided very poor humoral responses to LcrV (Figure 2 and Figure 3). A similar phenomenon was observed previously during the testing of several other *Yersinia*-based vaccine candidate strains in mice [42,43,44]. These data make us question the protective role for LcrV antigen alone in a live *Yersinia*-based vaccine setting as LcrV may not be expressed well at the place of replication of the vaccine strain or may interfere with other antigens produced by the *Yersinia* carrier. Another disadvantage is that the formulation of commercial lyophilized LPV contains a significant portion of dead bacteria (up to 70% and more) [6]. Moreover, the conditions of growth of the vaccine strain during LPV preparation at ambient temperature prevent the efficient expression of LcrV, and the entire Type 3 Secretion System (T3SS) as a whole for this matter. In contrast, LcrV expressed forcefully in other live carriers, such as adenovirus and other viral platforms, *Lactococcus lactis*, and plants, provided a robust immune response and protection [7,15,28,45,46,47,48]. Nevertheless, LcrV is expressed well in vitro by the *Y. pestis* EV line NIIEG, and its sequence (Figure S11, Supplementary Materials) is identical to that of fully virulent *Y. pestis* strains, such as CO92 (Acc. No. in NCBI AL117189.1), indicating that the LcrV still has protective potential in LPV. Indeed, in silico analysis predicted nine linear B-cell epitopes in LcrV molecule, five of which possessed antigenic activity, although only two of them, namely, PQTTIQVDGSEKKIV and GNLKNSYSYNKDNNELSHFATTCSDKSRP (numbers 8 and 9, respectively), were identified as nonallergens (Table 1). Similarly to the F1 antigen, these two epitopes were surface-exposed in LcrV (Figure S10, Supplementary Materials) indicating a possibility of priming the host immune system and inducing a proper immune response in vaccines. On the other hand, the relatively large size of epitope 9 (29 amino acids) may not be optimal for recognition by the immune system of vaccinees. Unfortunately, the predicted in silico epitopes for LcrV could not be verified experimentally, as the response to this antigen in vaccinees was poor. Nevertheless, the immunoreactivity of these epitopes was demonstrated previously in a mouse model [49,50,51]. In addition, in vitro LcrV stimulus increased the production of IL-10 in the A-Total group of vaccinees (Figure 2) suggesting a certain long-lasting T-cell immune response to this antigen.

Finally, there was no specific Th17 immune response detected to both F1 and LcrV in vaccinees (Figure 2), which indirectly corresponded to the absence of marked levels of F1-specific IgA in the sera of vaccinees (Figure S6, Supplementary Materials). This was different for the Pla antigen, which revealed strong Th17 polarization in donors vaccinated with LPV [22].

## 5. Conclusions

Our study provided insights into the role of the two main virulence proteins of *Y. pestis*, F1 and LcrV, in immunity to plagues induced after human immunization with live plague vaccine. Using the computational vaccinology tools, the B-cell epitopes of these antigens were mapped, followed by their experimental validation for the F1 antigen using sera from donors vaccinated with LPV. Five epitopes with reliable characteristics (antigenic, nontoxic, and nonallergenic), three for F1 and two for LcrV, were selected as promising vaccine targets and biomarkers or correlates of early and long-lasting protection acquired after vaccination with LPV. These data could be useful for the development of effective and safe next-generation vaccines against plague. This general approach could be valuable for the rational design of vaccines against other neglected and novel emerging infections with high pandemic potency.

## Figures and Tables

**Table 1:** Linear B-cell epitopes of F1 and LcrV predicted in silico.

Antigen	Epitope Number	B-cell Epitope ^1^	Position	Number of Residues	Antigenicity ^2^		Allergenicity ^2^	Toxicity ^3^	3D Structure
					Antigenicity Scores	Status			
F1	1	ADLTASTTATATLVE	22–36	15	0.7645	Antigen	Probable allergen	No	Figure S10A, Supplementary Materials
	2	KEGAPITIMDNGNIDT	45–60	16	0.5891	Antigen	Nonallergen	No	
	3	DAAGDPM	85–91	7	0.6144	Antigen	Nonallergen	No	
	4	TSQDGNNH	96–103	8	1.7902	Antigen	Probable allergen	No	
	5	KDSRDFDI	112–119	8	1.7530	Antigen	Probable allergen	No	
	6	NGENLVGDDVVLATG	124–138	15	0.6398	Antigen	Allergen	No	
	7	SKGGKLAAGKYTD	149–161	13	1.1808	Antigen	Nonallergen	No	
LcrV	1	GSSVLEELVQLVKDKNID	28–45	18	−0.0587	Nonantigen	Nonallergen	No	Figure S10A, Supplementary Materials
	2	KYDPRKDSEVFANRVITDDIELLKK	49–73	25	0.5326	Antigen	Allergen	No	
	3	AYFLPEDAILKGGHYDNQLQN	76–96	21	0.3679	Nonantigen	Nonallergen	No	
	4	ESSPNTQ	106–112	7	1.6615	Antigen	Allergen	No	
	5	NKHLSSSGT	175–183	9	1.0585	Antigen	Allergen	No	
	6	IHDKS	186–190	5	N/A	N/A	Allergen	No	
	7	MDKNLYGYTDEEIFKAS	194–210	17	0.3717	Nonantigen	Nonallergen	No	
	8	PQTTIQVDGSEKKIV	220–234	15	1.0058	Antigen	Nonallergen	No	
	9	GNLKNSYSYNKDNNELSHFATTCSDKSRP	251–279	29	0.6307	Antigen	Nonallergen	No	

^1^ B-cell epitopes were predicted using ElliPro (http://tools.iedb.org/ellipro/) [23]; ^2^ the VaxiJen v2.0 server [26] (http://www.ddg-pharmfac.net/vaxijen/VaxiJen/VaxiJen.html) and AllerTOP [27] v. 2.0 (http://www.ddg-pharmfac.net/AllerTOP) services were used for prediction of antigenicity and allergenicity; ^3^ the ToxinPred (http://crdd.osdd.net/raghava/toxinpred/) [25] web server was used to predict the toxicity of the peptides. N/A—not available; 3D—three-dimensional.

**Table 2:** Conformational B-cell epitopes of F1 and LcrV predicted in silico.

Antigen	Epitope #	B-cell Epitope Residues and Position ^1^	Number of Residues	Score	3D Structure
F1	1	B:A22, B:D23, B:L24, B:T25, B:A26, B:S27, B:T28, B:T29, B:A30, B:T31, B:A32, B:T33, B:L34, B:V35, B:E36, B:P37	16	0.849	Figure S10B, Supplementary Materials
	2	B:K45, B:E46, B:G47, B:A48, B:P49, B:I50, B:T51, B:I52, B:M53, B:D54, B:N55, B:G56, B:N57, B:I58, B:D59, B:T60, B:L62, B:T96, B:S97, B:Q98, B:D99, B:G100, B:N101, B:N102, B:H103, B:S149, B:K150, B:G151, B:G152, B:K153, B:L154, B:A155, B:A156, B:G157, B:K158, B:Y159, B:T160, B:D161	38	0.672	
	3	B:G75, B:T77, B:S78, B:T79, B:D85, B:A86, B:A87, B:G88, B:D89, B:P90, B:M91, B:Y92, B:K112, B:D113, B:S114, B:R115, B:D116, B:F117, B:D118, B:I119, B:S120, B:P121, B:K122, B:N124, B:G125, B:E126, B:N127, B:L128, B:V129, B:G130, B:D131, B:D132, B:V133, B:V134, B:L135	35	0.567	
LcrV	1	A:N175, A:K176, A:H177, A:L178, A:S179, A:S180, A:S181, A:G182, A:T183, A:I186, A:H187, A:D188, A:K189, A:G251, A:N252, A:L253, A:K254, A:N255, A:S256, A:S258, A:Y259, A:N260, A:K261, A:D262, A:N263, A:N264, A:E265, A:L266, A:S267, A:H268, A:F269, A:A270, A:T271, A:T272, A:C273, A:S274, A:D275, A:K276, A:S277, A:R278, A:P279	41	0.766	Figure S10B, Supplementary Materials
	2	A:K49, A:Y50, A:D51, A:P52, A:R53, A:K54, A:D55, A:S56, A:E57, A:V58, A:F59, A:A60, A:N61, A:R62, A:D148	15	0.741	
	3	A:N192, A:D195, A:K196, A:N197, A:L198, A:G200, A:Y201, A:T202, A:D203, A:E204, A:E205, A:I206, A:F207, A:K208, A:A209, A:S210, A:P220, A:Q221, A:T222, A:T223, A:I224, A:Q225, A:V226, A:D227, A:G228, A:S229, A:E230, A:K231, A:K232, A:I233, A:V234, A:S235	32	0.729	
	4	A:G28, A:S29, A:S30, A:V31, A:L32, A:E33, A:E34, A:V36, A:Q37, A:L38, A:V39, A:K40, A:D41, A:K42, A:N43, A:I44, A:D45, A:V63, A:I64, A:T65, A:D66, A:D67, A:I68, A:E69, A:L70, A:K72, A:K73, A:A76, A:F78, A:L79, A:P80, A:E81, A:D82, A:A83, A:I84, A:L85, A:K86, A:G87, A:G88, A:H89, A:Y90, A:D91, A:N92, A:Q93, A:L94, A:Q95, A:N96, A:E106, A:S108, A:P109, A:N110, A:T111, A:Q112	53	0.689	
	5	A:D313, A:S314, A:R318	3	0.67	

^1^ B-cell epitopes were predicted using ElliPro (http://tools.immuneepitope.org/toolsElliPro/) [23].

## Supplementary Materials

The following are available online at https://www.mdpi.com/2076-393X/8/4/698/s1: Figure S1. Analysis of association between either cytokine secretion or proliferative response and the number of LPV vaccinations. PBMCs from immunized donors (*n* = 18) were stimulated with recombinant F1 or LcrV (both at 5 μg/mL), and supernatants were analyzed for IFN-γ, TNF-α, IL-4, IL-10, and IL-17A levels. The correlation was estimated using Spearman’s rank correlation coefficient. The stimulation index (SI) was calculated against unstimulated cells. No correlation was revealed for all tested cytokines, as well as between proliferation (SI) and post-immunization time (*p* > 0.05); Figure S2. Analysis of association between cytokine secretion or proliferation and post vaccination time in years. PBMCs from immunized donors (*n* = 18) were stimulated with recombinant F1 or LcrV (both at 5 μg/mL), and supernatants were analyzed for IFN-γ, TNF-α, IL-4, IL-10, and IL-17A levels. The correlation was calculated with Spearman’s rank correlation coefficient. The SI was calculated against unstimulated cells. No correlation was revealed for all tested cytokines, as well as between proliferation (SI) and post-immunization time (*p* > 0.05); Figure S3. Serum antibody titers to F1 and LcrV antigens revealed by ELISA in sera obtained from vaccinees (groups A-Total, A-RV, and A-EV) and naïve donors (group B). Difference between groups was analyzed by Mann–Whitney test; ** *p* < 0.01; Figure S4. Analysis of correlations between percent of positive antisera revealed by immunoblot technique to either F1 or LcrV antigens: (**a**) number of vaccinations with LPV; (**b**) post vaccination time. Spearman’s rank correlation coefficient and *p*-value were calculated. No significant correlations were detected in all cases; Figure S5. Analysis of correlations between number of vaccinations with LPV or post-vaccination time: (**a**) percent of positive antisera to F1 revealed by ELISA; (**b**) anti-F1 antibody titers. Spearman’s rank correlation coefficient and *p*-value were calculated. No significant correlations were detected in all cases; Figure S6. Distribution of classes and subclasses of anti-F1 immunoglobulins in vaccinees (**A**) and unvaccinated donors (**B**) detected in ELISA. Sera from 11 of F1-seropositive vaccinated and three unvaccinated donors were used for analysis; Figure S7. Distribution of classes and subclasses of anti-F1 immunoglobulins in vaccinees (**A**) and unvaccinated donors (**B**) detected by immunoblotting technique. Sera from 15 of F1-seropositive vaccinated donors and one unvaccinated donor were used for analysis; Figure S8. Receiver operating characteristic (ROC) analysis for anti-F1 serum antibody titers in group A-RV versus group B. The ROC curve showed AUC = 0.7388 ± 0.09469 with *p* < 0.05 indicating that anti-F1 antibody titers could discriminate recently vaccinated donors from unvaccinated controls. However, the maximum Youden’s index was only 42.86 in case of cutoff = 150 with absolute specificity (100%), but low sensitivity (42,86%). AUC: area under the curve; SE: standard error; CI: confidence intervals; Figure S9. Receiver operating characteristic (ROC) analysis for anti-F1 serum antibody titers in group A-Total versus group B. In this case, anti-F1 antibody titers did not discriminate among all donors who received vaccination with the LPV and naïve control volunteers without LPV immunization (*p* > 0.05). AUC: area under the curve; Figure S10. 3D ribbon diagrams modeling F1 and LcrV proteins on the basis of the relevant original amino-acid sequences. (**A**) B-cell linear epitopes residues of the F1 and LcrV. (**B**) B-cell conformational epitopes residues of the F1 and LcrV; Figure S11. Alignments of the nucleotide sequences of *lcrV* gene from *Y. pestis* CO92 (Acc. No. in NCBI AL117189.1) and the commercial strain of the *Y. pestis* EV line NIIEG (Acc. No. in NCBI MT952719); Table S1. Library of 27 overlapping 17-mer synthetic peptides designed based on the 170-amino-acid sequence of the F1 protein from *Y. pestis* CO92 (GenBank accession No. NP_395430.1). The peptides were made with >95% purity (GenScript, Piscataway, NJ, USA) and stored in aliquots (stock concentration of 10 mg/mL) at −80 °C; Table S2. Human antibody response measured by ELISA to F1 and LcrV in vaccinated and unvaccinated groups.
