# Supplementary material for: Yersinia pestis Antigen F1 but Not LcrV Induced Humoral and Cellular Immune Responses in Humans Immunized with Live Plague Vaccine—Comparison of Immunoinformatic and Immunological Approaches
Source: Vaccines (Basel). 2020 Nov 19;8(4):698. doi: 10.3390/vaccines8040698 (PMC7712656; doi:10.3390/vaccines8040698)
Supplement: Supplementary file 1 [file vaccines-08-00698-s001.pdf]

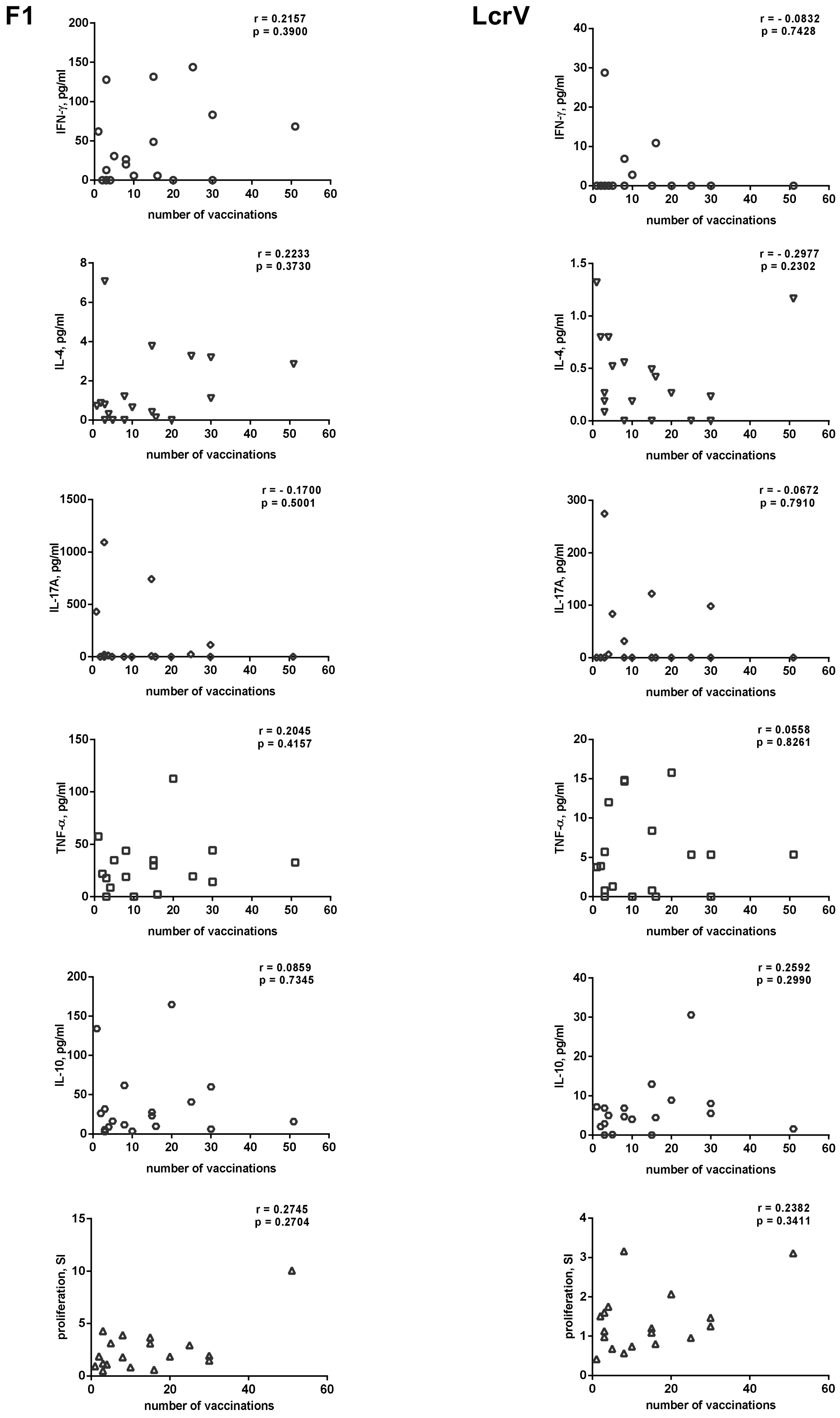

F1
LcrV
r = 0.2157
p = 0.3900
IFN-γ, pg/ml
number of vaccinations
r = - 0.0832
p = 0.7428
IFN-γ, pg/ml
number of vaccinations
r = 0.2233
p = 0.3730
IL-4, pg/ml
number of vaccinations
r = - 0.2977
p = 0.2302
IL-4, pg/ml
number of vaccinations
r = - 0.1700
p = 0.5001
IL-17A, pg/ml
number of vaccinations
r = - 0.0672
p = 0.7910
IL-17A, pg/ml
number of vaccinations
r = 0.2045
p = 0.4157
TNF-α, pg/ml
number of vaccinations
r = 0.0558
p = 0.8261
TNF-α, pg/ml
number of vaccinations
r = 0.0859
p = 0.7345
IL-10, pg/ml
number of vaccinations
r = 0.2592
p = 0.2990
IL-10, pg/ml
number of vaccinations
r = 0.2745
p = 0.2704
proliferation, SI
number of vaccinations
r = 0.2382
p = 0.3411
proliferation, SI
number of vaccinations

Figure S1. Analysis of association between either cytokine secretion or proliferative response and a number of vaccinations with the LPV. PBMCs from immunized donors (n=18) were stimulated with recombinant F1 or LcrV (both at 5 ◎g/ml), and supernatants were analyzed for IFN-γ, TNF-α, IL-4, IL-10, and IL-17A levels. The correlation was estimated using Spearman's Rank Correlation coefficient. The Stimulation index (SI) was calculated against unstimulated cells. No correlation was revealed for all tested cytokines, as well as between proliferation (SI) and post-immunization time ($p>0.05$).

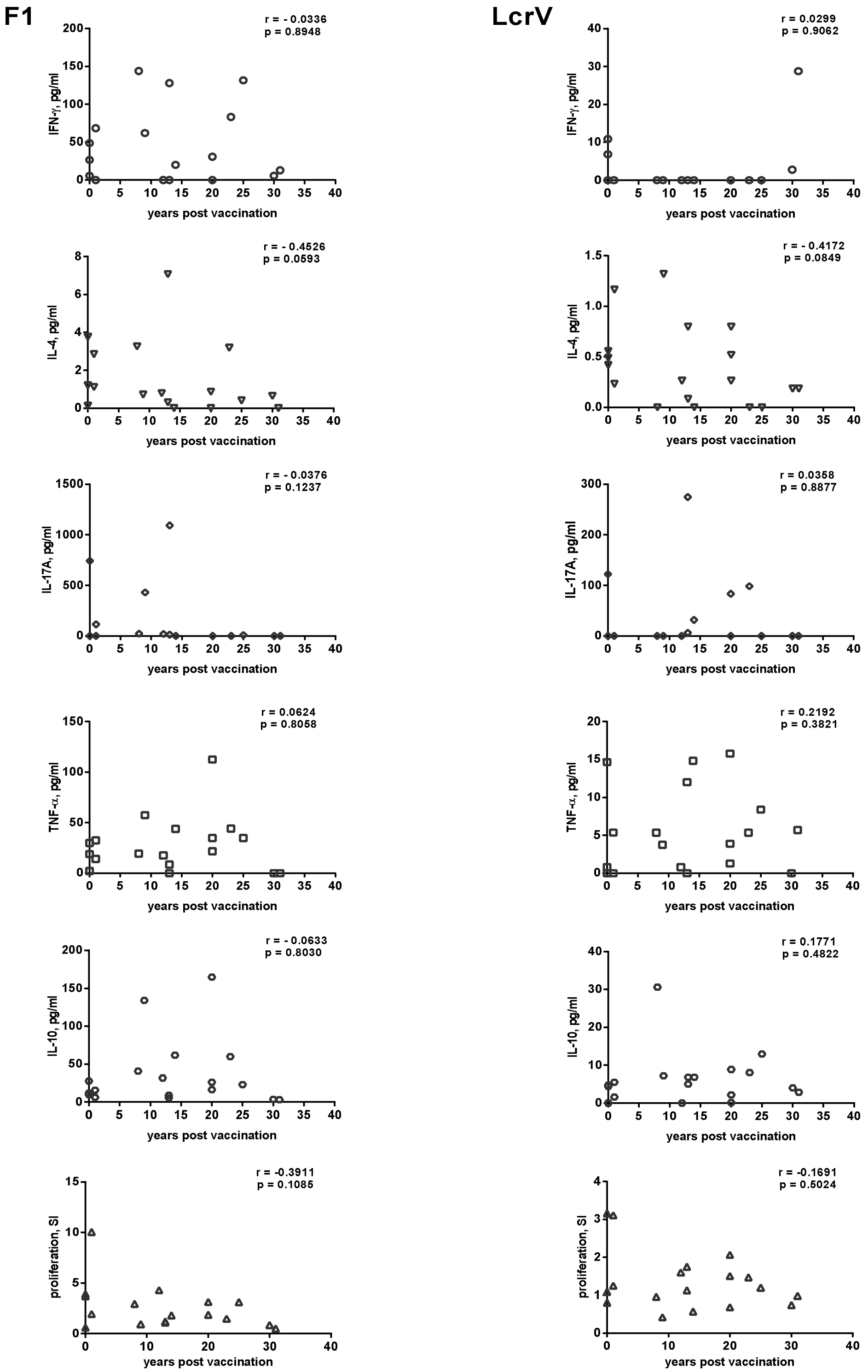

Figure S2. Analysis of association between cytokine secretion or proliferation and post vaccination time in years. PBMCs from immunized donors (n=18) were stimulated with recombinant F1 or LcrV (both at 5 μg/ml), and supernatants were analyzed for IFN-γ, TNF-α, IL-4, IL-10, and IL-17A levels. The correlation was calculated with Spearman's Rank Correlation coefficient. The SI was calculated against unstimulated cells. No correlation was revealed for all tested cytokines, as well as between proliferation (SI) and post-immunization time ($p>0.05$).

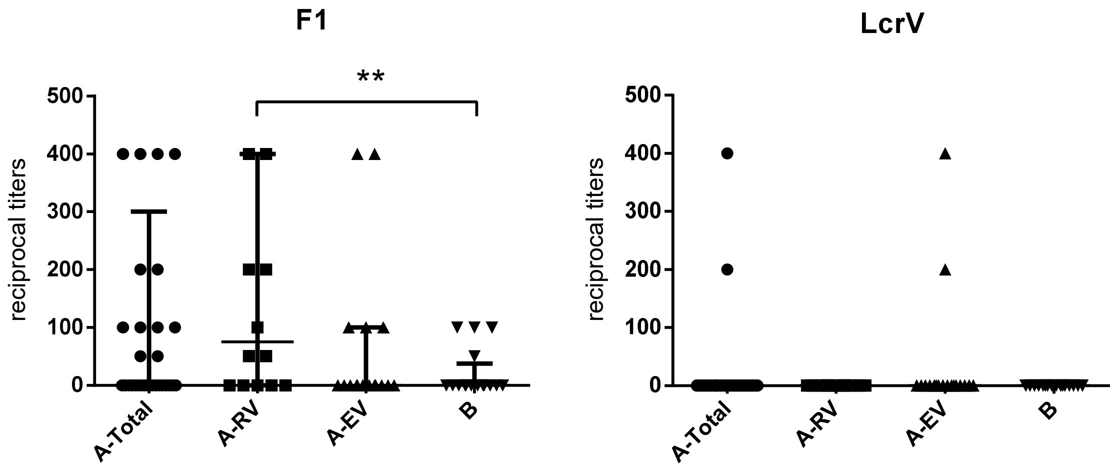

Figure S3. Serum antibody titers to F1 and LcrV antigens revealed by ELISA in sera obtained from vaccinees (groups A-Total, A-RV, A-EV) and naïve donors (group B). Difference between groups was analyzed by Mann-Whitney test; **$p < 0.01$.

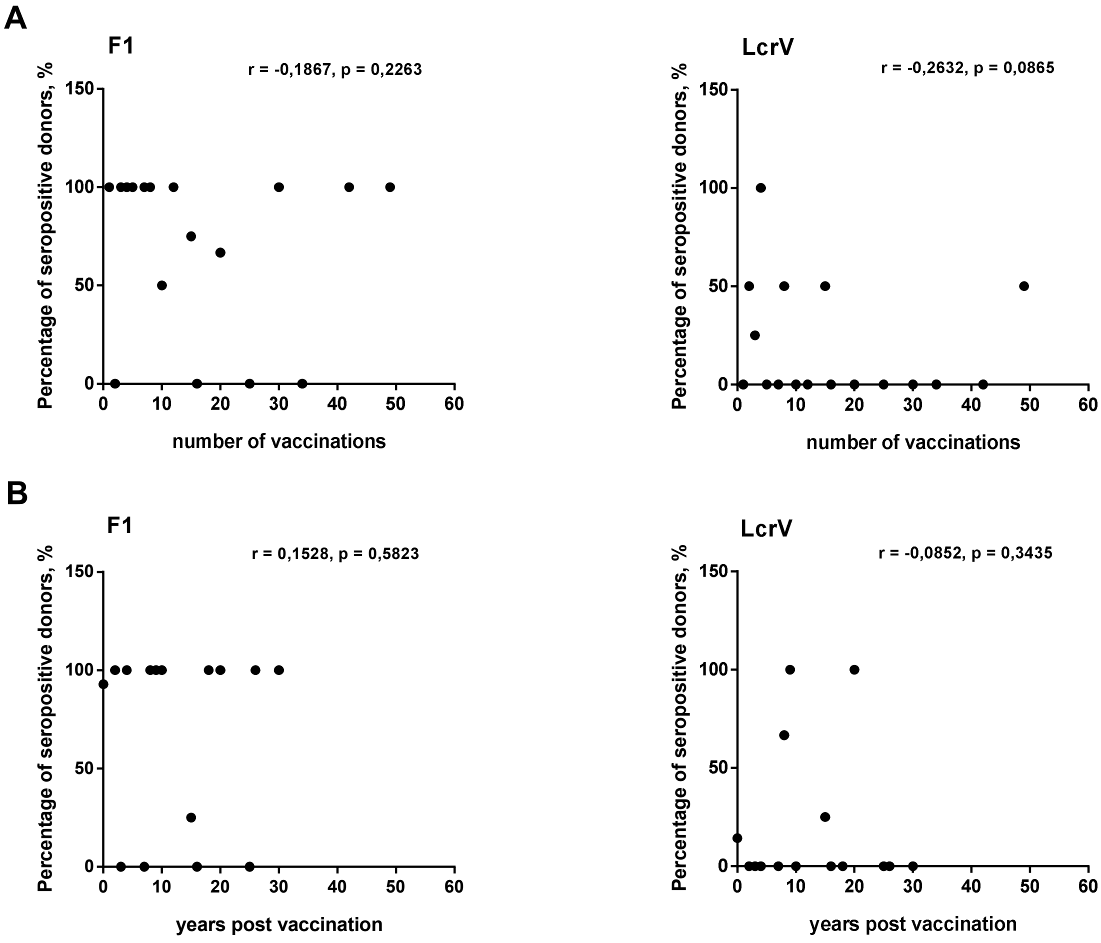

Figure S4. Analysis of correlations between percent of positive antisera revealed by immunoblot technique to either F1 or LcrV antigens: (a) number of vaccinations with LPV; (b) post vaccination time. Spearman's Rank Correlation Coefficient and p-value were calculated. No significant correlations were detected in all cases.

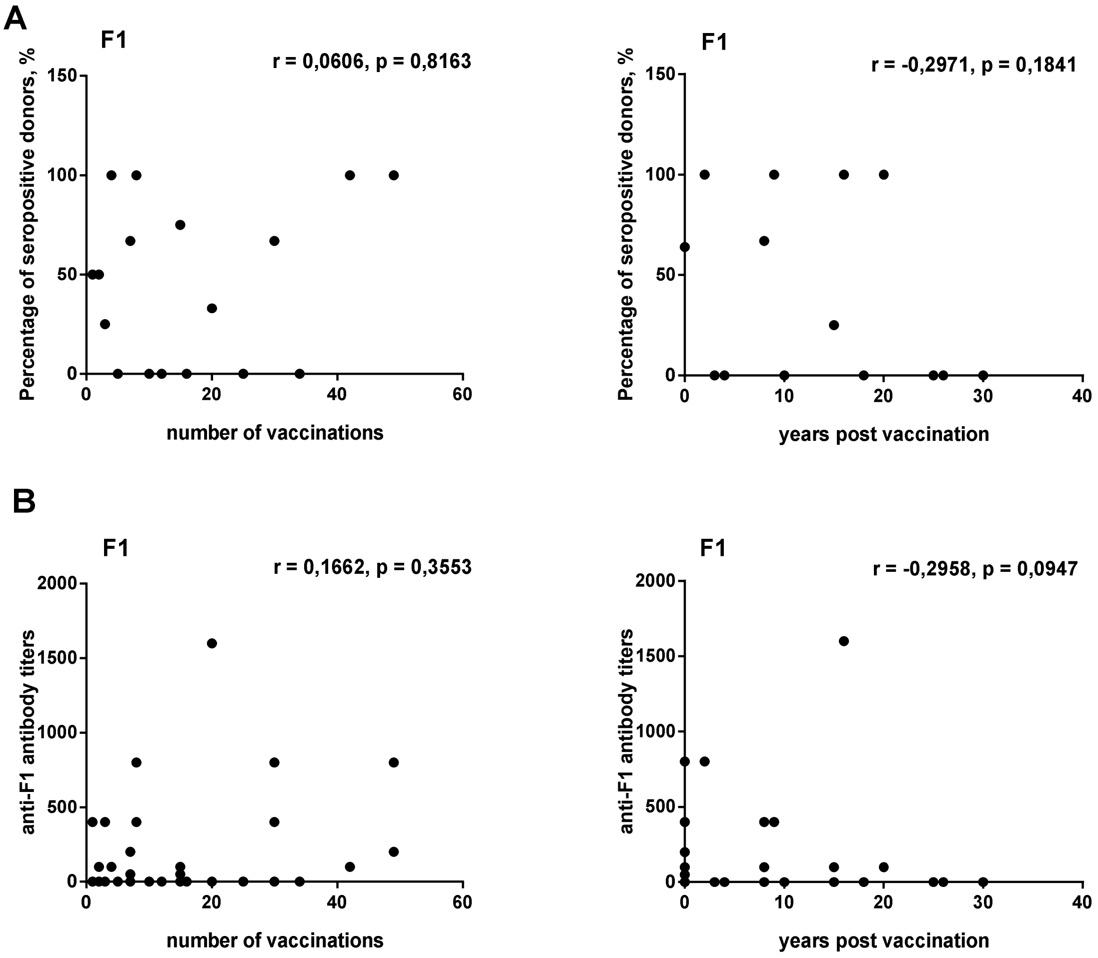

Figure S5. Analysis of correlations between number of vaccinations with LPV or post vaccination time: (a) percent of positive antisera to F1 revealed by ELISA; (b) anti-F1 antibody titers. Spearman's Rank Correlation Coefficient and p-value were calculated. No significant correlations were detected in all cases.

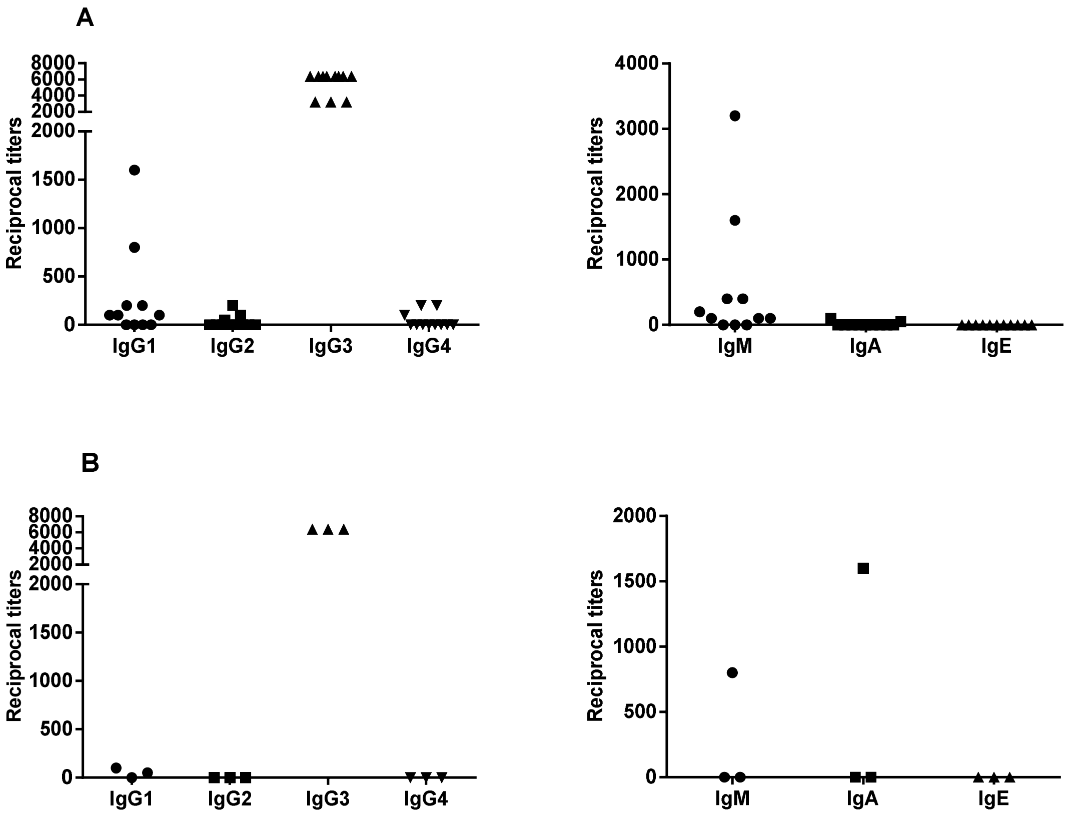

Figure S6. Distribution of classes and subclasses of anti-F1 immunoglobulins in vaccinees (A) and unvaccinated donors (B) detected in ELISA. Sera from 11 of F1-seropositive vaccinated and 3 unvaccinated donors were used for analysis.

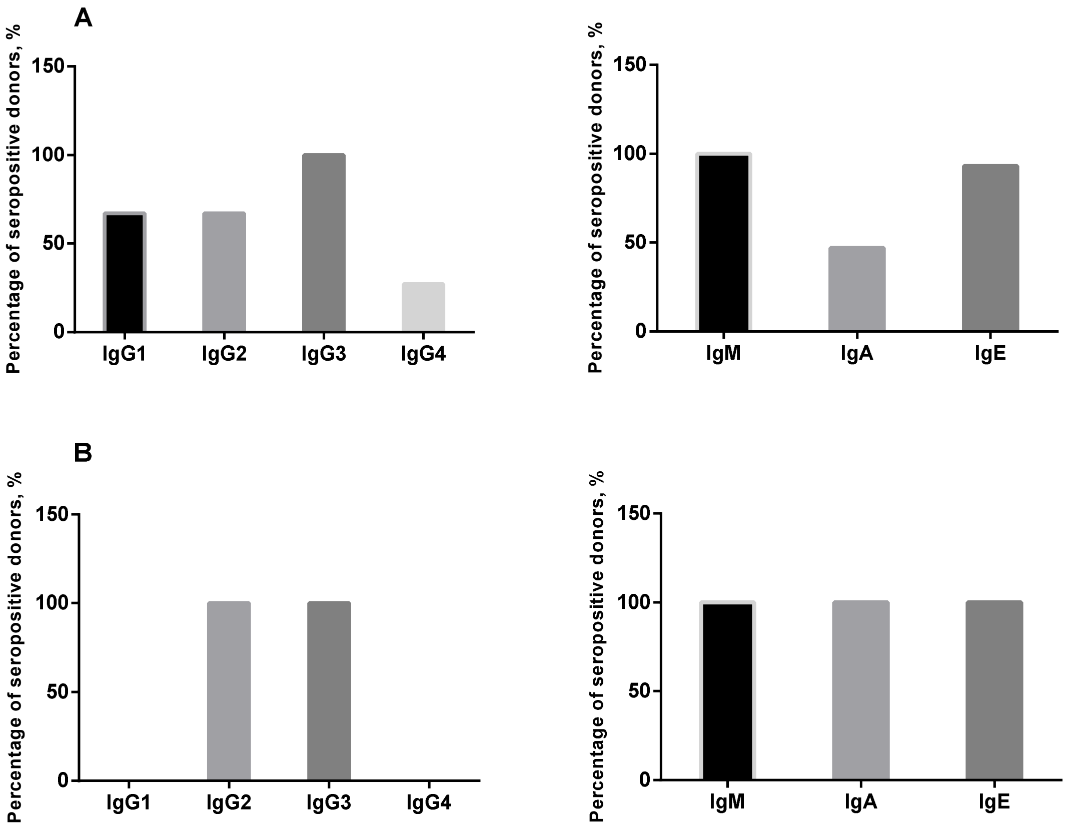

Figure S7. Distribution of classes and subclasses of anti-F1 immunoglobulins in vaccinees (A) and unvaccinated donors (B) detected by immunoblotting technique. Sera from 15 of F1-seropositive vaccinated donors and 1 unvaccinated donor were used for analysis.

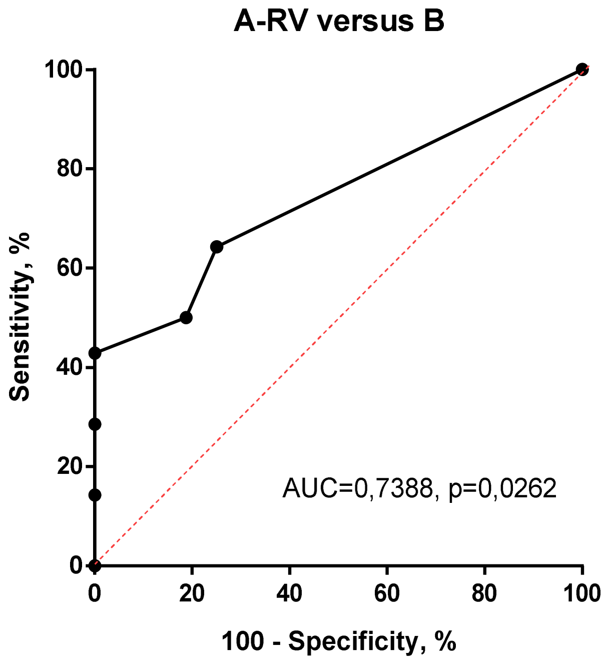

Area under the curve				Criterion values in dependence on the cutoff value					
AUC	SE	95% CI	p value	Cutoff	Sensitivity, %	95% CI	Specificity, %	95% CI	Youden's index
0,7388	0,0947	0,5532 - 0,9245	0,0262	> 25.00	64,29	35,14-87,24	75,00	47,62-92,73	39,29
				> 75.00	50,00	23,04-76,96	81,25	54,35-95,95	31,25
				> 150.0	42,86	17,66-71,14	100,0	79,41-100,0	42,86
				> 300.0	28,57	8,389 - 58,10	100,0	79,41-100,0	28,57
				> 600.0	14,29	1,779-42,81	100,0	79,41-100,0	14,29

Figure S8. Receiver operating characteristic (ROC) analysis for anti-F1 serum antibody titers in group A-RV versus group B. ROC curve showed AUC=0,7388±0,09469 with $p<0.05$ indicating that anti-F1 antibody titers could discriminate recently vaccinated donors from unvaccinated controls. However, the maximum Youden's index was only 42,86 in case of cutoff = 150 with absolute specificity (100%), but low sensitivity (42,86%). AUC: area under the curve; SE: standard error; CI: confidence interval.

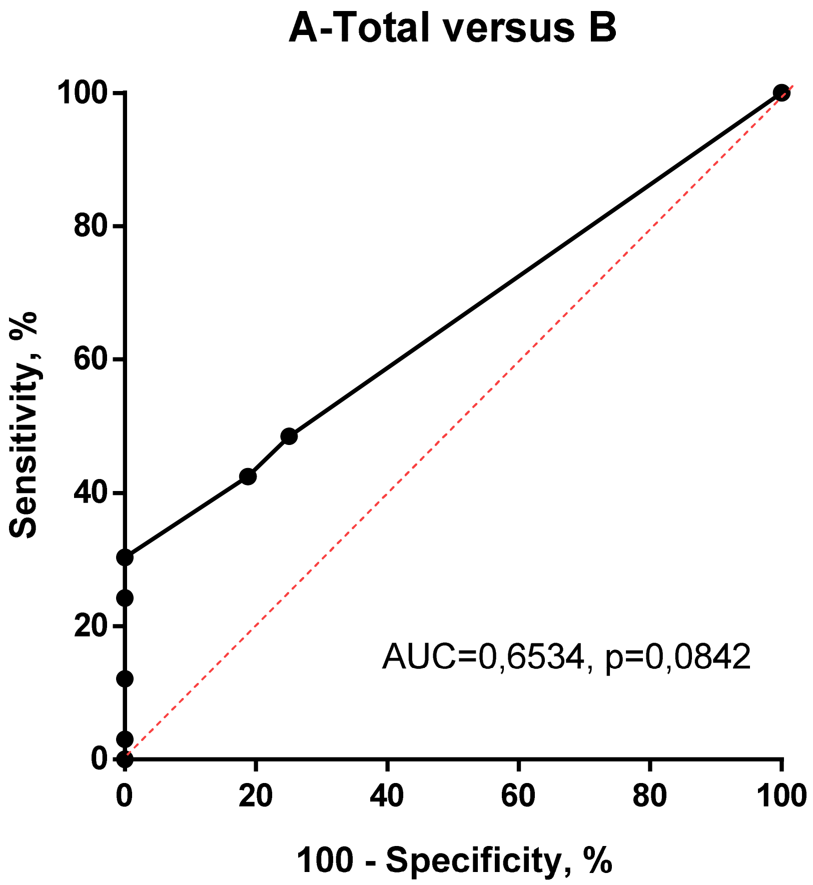

Figure S9. Receiver operating characteristic (ROC) analysis for anti-F1 serum antibody titers in group A-Total versus group B. In this case, anti-F1 antibody titers were not discriminating between all donors who ever have been received vaccination with the LPV and naïve control volunteers without LPV immunization ($p>0.05$). AUC: area under the curve.

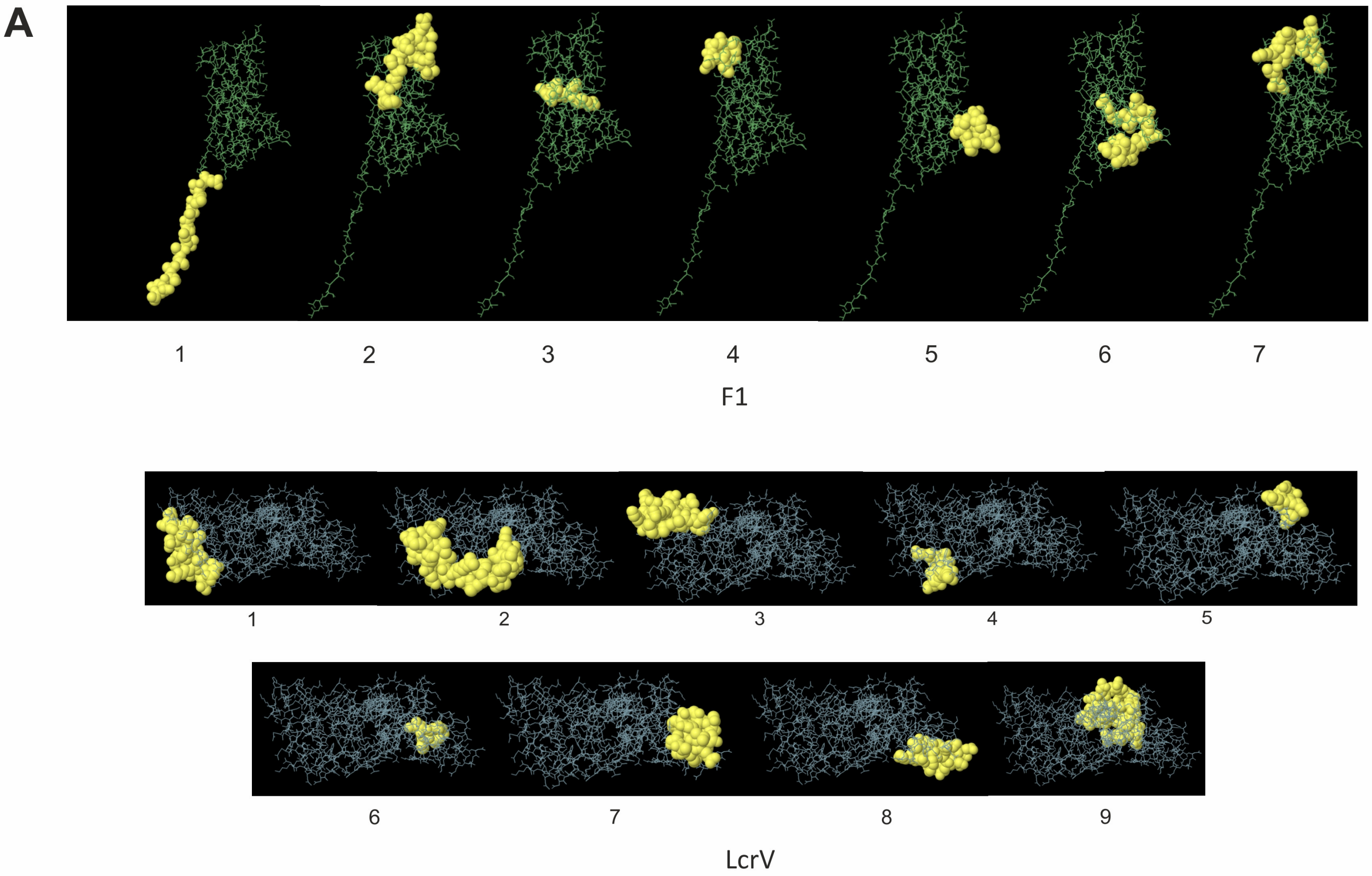
A
1
2
3
4
5
6
7
F1
1
2
3
4
5
6
7
8
9
LcrV

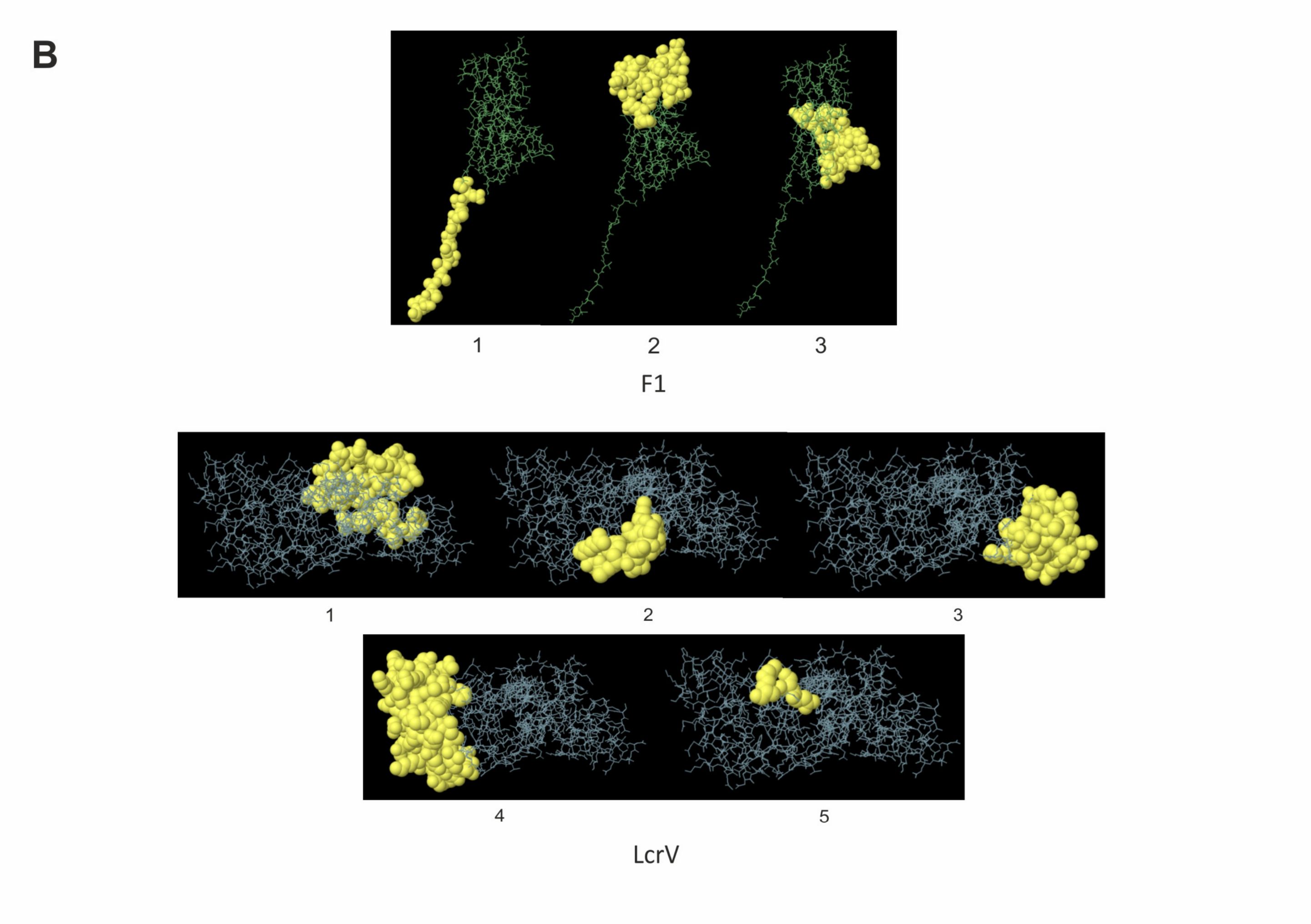

Figure S10. Structural 3D ribbon diagrams modeling of F1 and LcrV proteins based on the relevant original amino acid sequences. (A) B-cell linear epitopes residues of the F1 and LcrV. (B) B-cell conformational epitopes residues of the F1 and LcrV.

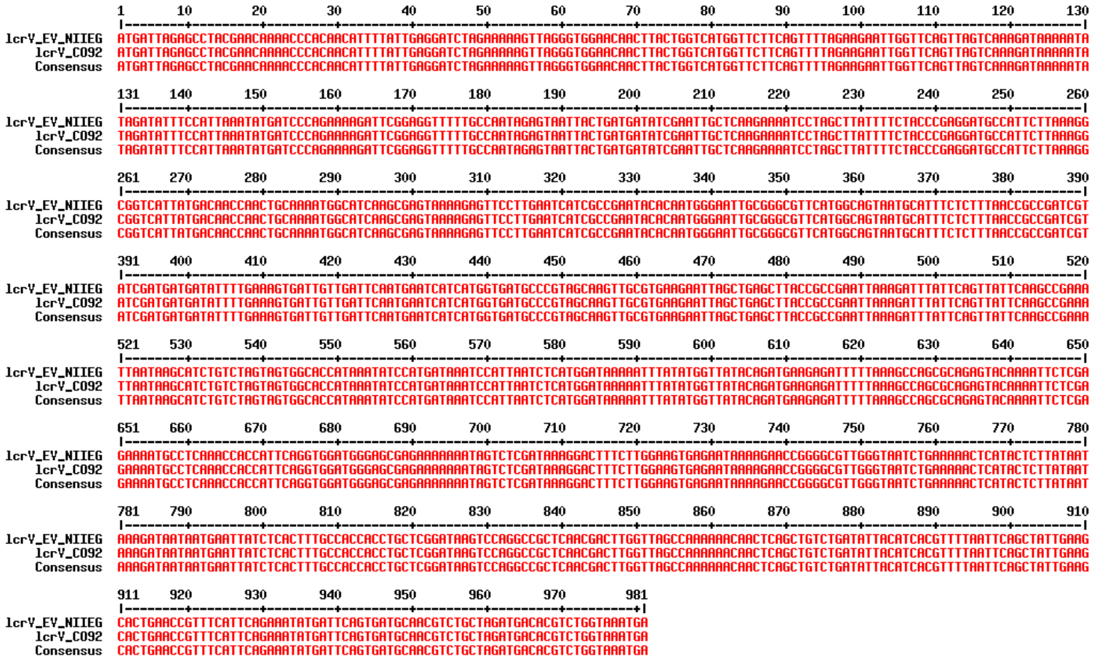

Figure S11. Alignments of the nucleotide sequences of *lcrV* gene from: (A) the *Y. pestis* CO92 (Acc. No. in NCBI AL117189.1) and (B) the commercial strain of the *Y. pestis* EV line NIIEG (Acc. No. in NCBI MT952719).

S1 Table. Library of 27 overlapping 17-mer synthetic peptides designed based on 170 amino acid sequence of the F1 protein from Y. pestis CO92 (GenBank accession No. NP_395430.1). The peptides were made with >95% purity (GenScript, Piscataway, NJ) and stored in aliquots (stock concentration of 10 mg/ml) at -80°C.

Peptide ID	Peptide length	Sequence
1. F1_1-17 (signal)	17	MKKISSVIAIALFGTIA
2. F1_7-24 (signal)	17	VIAIALFGTIATANAAD
3. F1_13-29	17	FGTIATANAADLTASTT
4. F1_19-35	17	ANAADLTASTTATATLV
5. F1_25-41	17	TASTTATATLVEPARIT
6. F1_31-47	17	TATLVEPARITLTYKEG
7. F1_37-53	17	PARITLTYKEGAPITIM
8. F1_43-59	17	TYKEGAPITIMDNGNID
9. F1_49-65	17	PITIMDNGNIDTELLVG
10. F1_55-71	17	NGNIDTELLVGTLTLGG
11. F1_61-77	17	ELLVGTLTLGGYKTGTT
12. F1_67-83	17	LTLGGYKTGTTSTSVNF
13. F1_73-89	17	KTGTTSTSVNFTDAAGD
14. F1_79-95	17	TSVNFTDAAGDPMYLTF
15. F1_85-101	17	DAAGDPMYLTFTSQDGN
16. F1_91-107	17	MYLTFTSQDGNNHQFTT
17. F1_97-113	17	SQDGNNHQFTTKVIGKD
18. F1_103-119	17	HQFTTKVIGKDSRDFDI
19. F1_109-125	17	VIGKDSRDFDISPKVNG
20. F1_115-131	17	RDFDISPKVNGENLVGD
21. F1_121-137	17	PKVNGENLVGDDVVLAT
22. F1_127-143	17	NLVGDDVVLATGSQDFF
23. F1_133-149	17	VVLATGSQDFFVRSIGS
24. F1_139-155	17	SQDFFVRSIGSKGGKLA
25. F1_145-161	17	RSIGSKGGKLAAGKYTD
26. F1_151-167	17	GGKLAAGKYTDAVTVTV
27. F1_157-170	14	GKYTDAVTVTVSNQ

Table S2. Human antibody response measured by ELISA to F1 and LcrV in vaccinated and unvaccinated groups.

Antigen	Parameter	Donor's group			
		A-Total	A-RV	A-EV	B
F1	GMT (95% CI)	76,10 (46,73-123,9)	100,0 (45,62-219,2)	62,23 (31,89-121,5)	33,86 (25,07-45,73)
	Reciprocal titers, Min to Max	50-1600	50-800	100-1600	50-100
LcrV	GMT (95% CI)	29,09 (23,41-36,16)	25,00 (25,00-25,00)	32,73 (22,06-48,57)	25,00 (25,00-25,00)
	Reciprocal titers, Min to Max	200-400	-	200-400	-

[1] A-Total – all donors vaccinated with LPV, A-RV – recently vaccinated, A-EV – early vaccinated, B – naïve unvaccinated volunteers; GMT - geometric mean titers, CI – confidence interval, Min to Max – minimum to maximum, reciprocal titer limits. For GMT calculation titers below the limit of detection (< 50) were arbitrarily assigned a value of 25.
